# The lack of floater perception in eyes with asteroid hyalosis and its direct implications on laser vitreolysis

**DOI:** 10.1186/s40942-024-00601-0

**Published:** 2024-10-24

**Authors:** Elie Zaher, Yonatan Blumenthal, Eytan Z. Blumenthal

**Affiliations:** 1 Department of Ophthalmology, Rambam Health Care Campus, P.O.B 9602, Haifa, 31096 Israel; 2 Ruth and Bruce Rappaport Faculty of Medicine, Technion - Israel Institute of Technology, Haifa, Israel

**Keywords:** Asteroid hyalosis, Floaters, Vitreolysis, Myodesopsia, Umbra, Eclipse

## Abstract

**Purpose:**

To present a novel optical model explaining why the vast majority of patients with Asteroid Hyalosis (AH) do not perceive any floaters. This changes our understanding of floater perception and undermines the operation mode of YAG laser vitreolysis.

**Methods:**

Relying on a previously published model of floater perception based on astronomical equations of a solar eclipse, and on ultrasound images of the vitreous in three eyes with AH, we explain why such patients do not perceive floaters in spite of opaque bodies filling their entire vitreous, to the point of, in severe cases of AH, obscuring the fundus view during ophthalmoscopy.

**Main outcome measures:**

Developing an optical model of light rays that can quantify the maximal distance upon which a vitreous floater or opacity will cast a shadow on the retina.

**Results:**

Calculations using the proposed model demonstrated that with a 3 mm pupil, for a floater located between 1.5 mm and 2 mm from the retina, its shortest diameter must be > 215 microns and > 286 microns, respectively, to be perceived. Since AH floaters, based on ultrasound imaging, do not exist in the most peripheral 1.5 mm of the vitreous, it becomes understandable why these patients are asymptomatic.

**Conclusions:**

Based on the proposed model and our findings, we deduced that even large, degenerative floaters whose width is usually narrower than a large retinal vein (125 microns), must be located very close to the retina and hence are not the floaters that are aimed at when performing YAG laser vitreolysis. We speculate that in successful cases, YAG vitreolysis works by a different mechanism, most likely a shock wave that displaces floaters further away from the retina. Hence, vitreolysis might not necessarily require the laser be aimed at the floaters, as symptomatic floaters may be located in the outer 1.5–2.0 mm of the vitreous body, a very risky zone for YAG laser shots.

**Supplementary Information:**

The online version contains supplementary material available at 10.1186/s40942-024-00601-0.

Asteroid hyalosis (AH) is a condition in which multiple, small, yellow-white refractile particles present in the vitreous body [1]. AH was first described by Benson in 1894 and initially termed “Asteroid Hyalites”, due to its clinical appearance resembling stars (or asteroids) shining in the night sky [2, 3]. Seventy years later, it was renamed “Asteroid Hyalosis” due to the absence of inflammatory changes [3]. AH bodies are mobile and their diameter ranges between 25 and 125 microns [1, 4]. The opacities have a smooth spherical morphology and are composed of lipids complexed with calcium, phosphates, and oxygen [4, 5]. While the pathogenesis of AH is still not fully understood, it is thought to be a benign degenerative condition similar to cholelithiasis and nephrolithiasis [1, 5]. 

AH is mostly a unilateral condition [4, 6] with an estimated prevalence ranging between 0.8 and 2% in the general population, based on large epidemiological studies [6–9]. While many risk factors are associated with AH, older age is the only consistent statistically significant risk factor [6–9]. An inverse correlation between AH and posterior vitreous detachment (PVD) has been noted in several studies [4, 6, 9]. 

AH bodies can be demonstrated in multiple imaging modalities [10, 11]. In ultrasonographic B-scan, both diffuse and focal hyperechoic spots with considerable after movement are seen in the vitreous cavity, separated by a clear zone of vitreous between the posterior border of AH bodies and retinal surface [10, 12, 13]. 

Although AH may significantly impair Fundus visualization, it rarely causes any visual symptoms or affects patients’ vision [1, 4, 6, 14]. Several unproven hypotheses have been suggested to explain this phenomenon. The current leading hypothesis suggests that unlike the opacities in PVD, AH bodies have smooth surfaces and thus do not cause light scattering or optical aberrations [1, 15]. While smoothness affects how the light interacts with the object in terms of reflection and scattering, but as long as the object is opaque and positioned in the path of the light, it can still cast a shadow and thus be visually significant [16, 17]. Alternative, hypotheses suggest that AH bodies are located mainly in the anterior vitreous and thus have less impact on light reaching the fovea [1], or are sparsely distributed in the vitreous [10, 18, 19]. These hypotheses conflict with the obscuration of the fundus view of the examining ophthalmologist, as well as the far more noticeable presence of these opacities in contrast to other vitreous floaters, such as a Weiss-ring.

Understanding this phenomenon could have an impact on the management of patients with impaired vision due to vitreous opacities, while several treatment options such as vitrectomy and more recently laser vitreolysis have been tried [11, 20, 21]. 

In 1997, Serpetopoulos et al. proposed a theory explaining the gradual disappearance of symptoms in PVD, the most common cause of myodesopias (also known as floaters) [11, 22, 23]. His model was based on an analogy to the astronomical phenomenon of a solar eclipse where a spherical opaque body receives light from a source whose diameter is larger than its own and thus casts a conoid shadow [22]. In their model, the sun or light source is analogous to the pupil, the moon to the vitreous opacity, and the earth to the retina [22]. In 1998, with the help of an astronomy professor, they built a mathematical model and were able to calculate and estimate the size and density of the umbra (full shadow) casted by vitreous opacities of variable sizes and distances from the retina [24]. Three important determining factors were identified: (1) opacity’s distance from the retina, (2) diameter of the opacity, and (3) diameter of the pupil [22, 24]. The retinal area obscured by the shadow (area in which the photoreceptors ‘experience’ an eclipse) was found to increase with larger opacity size and decrease with increased opacity distance from retina, as well as with increased pupil diameter [22, 24]. Their conclusion was that in PVDs, as the detached vitreous moves further forward over time, the opacity’s distance from the retina increases and therefore perception of floaters gradually diminishes [22, 24]. 

In this article, relying on the Serpetopoulos model as well as using B-mode ultrasound imaging, we introduced an alternative explanation as to why AH bodies, despite being refractile, dense, and mobile, only rarely cause any visual symptoms.

## Methods

**Fig. 1:**
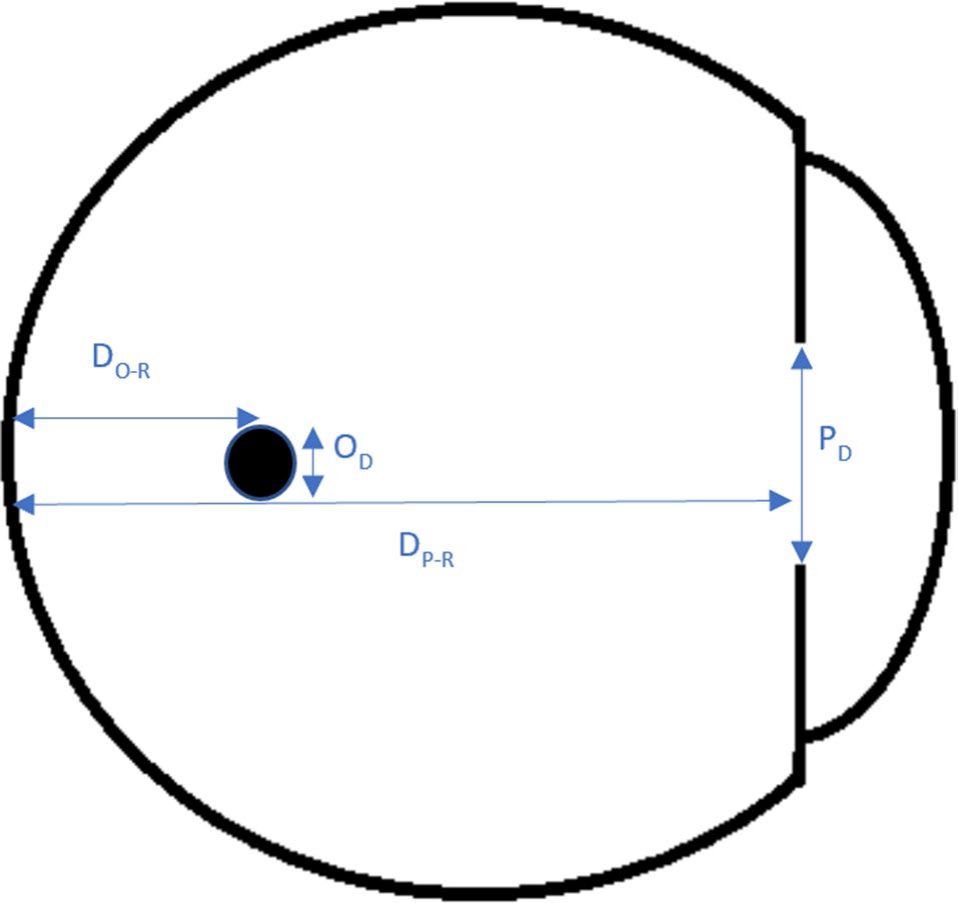
An eye model with an intravitreal opacity (black circle) and the relevant distances. P_D_: pupil diameter, O_D_: opacity diameter, D_P−R_: distance between pupil and retina, D_O−R_: distance between opacity and retina

**Fig. 2:**
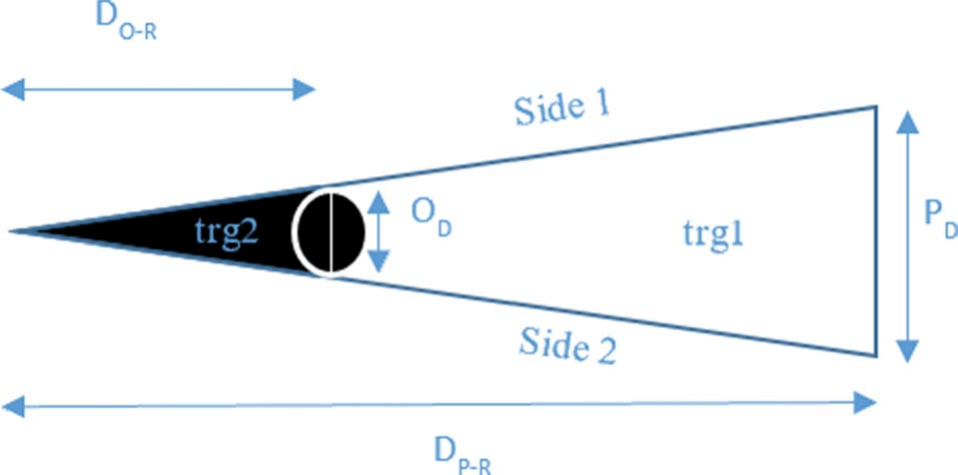
A geometrical model of the shadow casted by a spherical opacity. Side 1 & 2: light rays, black area: area of a shadow. P_D_: pupil diameter, O_D_: opacity diameter, D_P−R_: distance between pupil and retina, D_O−R_: distance between opacity and retina

**Fig. 3:**
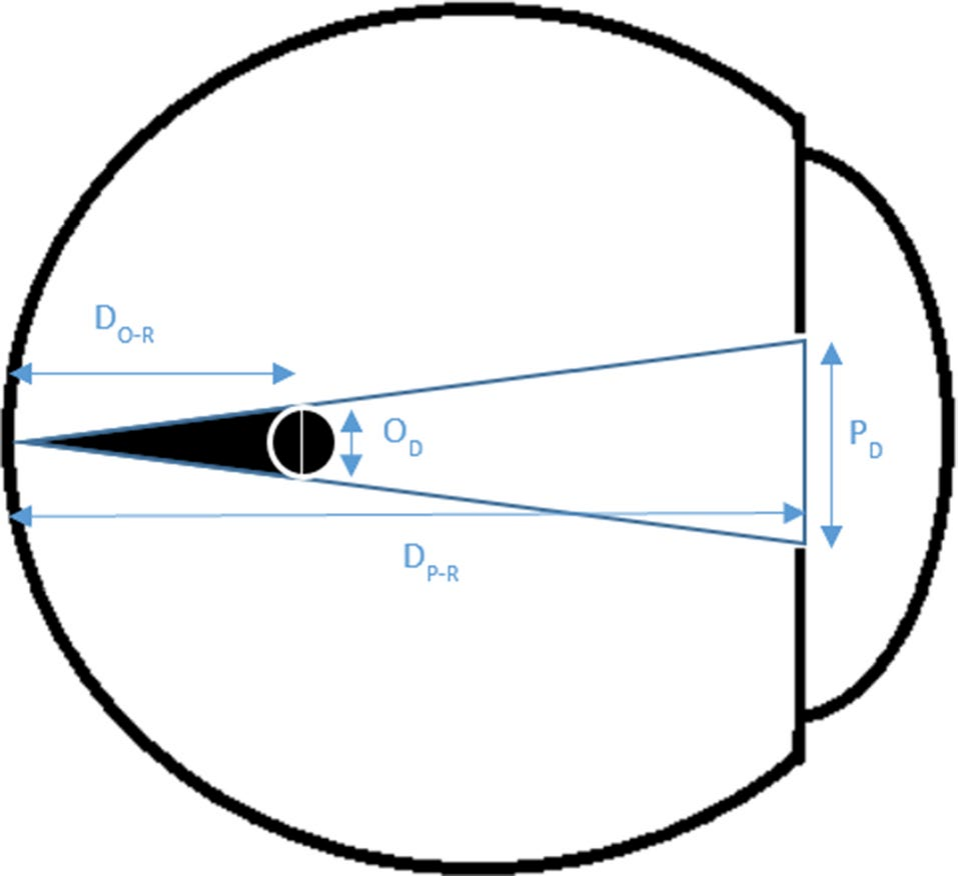
The triangle depicted in Fig. 2, as it pertains to the eye

In order to decide whether an opacity with a diameter similar to AH bodies casts a shadow on the retina, we relied on basic triangle similarity rules and a modified previously published model of floater perception based on astronomical equations of a solar eclipse [22, 24]. We assumed a pupil diameter of 3 mm (similar to the original model) [22, 24]; pupil to retina distance of 21 mm in an emetropic eye (similar to the original model); and various opacity diameters (independent variables). The opacity to retina distance was the dependent variable (Figs. 1, 2 and 3). Using the model and its derived equation (Eq. 1) we calculated, for each opacity diameter, the maximal distance from the retina where the tip of the umbra (dark cone) casts a shadow (touches) on a very small (minimal) area of the retina. In a separate analysis, assuming a fixed opacity diameter (125 microns), we calculated the maximal possible opacity distance from the retina (dependent variable) for different pupil diameters to cast a retinal shadow (Eq. 2).

1 $$\text{O}_{\text{D}}=(\text{P}_\text{D}/\text{D}_{\text{P}-\text{R}})\:^{*}\:\text{D}_{\text{O}-\text{R}}$$

P_D_: pupil diameter, O_D_: opacity diameter, D_P−R_: distance between pupil and retina, D_O−R_: distance between opacity and retina.

2 $$\text{D}_{\text{O}-\text{R}}=(\text{O}_\text{D}/\text{P}_{\text{D}})^{*}\:\text{D}_{\text{P}-\text{R}}$$

D_O-R_: maximal distance between opacity and retina, O_D_: opacity diameter, D_P-R_: distance between pupil and retina, P_D_: pupil diameter.

We then identified in Rambam’s Department of Ophthalmology (Haifa, Israel) ultrasound archive three images of 2 subjects who had been scanned and found to have AH. We measured the distance of the closest AH bodies to the retinas in these scans (i.e., the thickness of the AB-free most peripheral vitreous zone).

Based on the above calculations related to opacity size typical of AH [1, 4], we aimed to explain why AH bodies in AH do not cast a shadow on the retina and thus rarely cause any visual symptoms.

The Institutional Review Board (IRB) Committee at Rambam Health Care Campus ruled that approval was not required for this study and an exemption letter was granted.

## Results

**Fig. 4:**
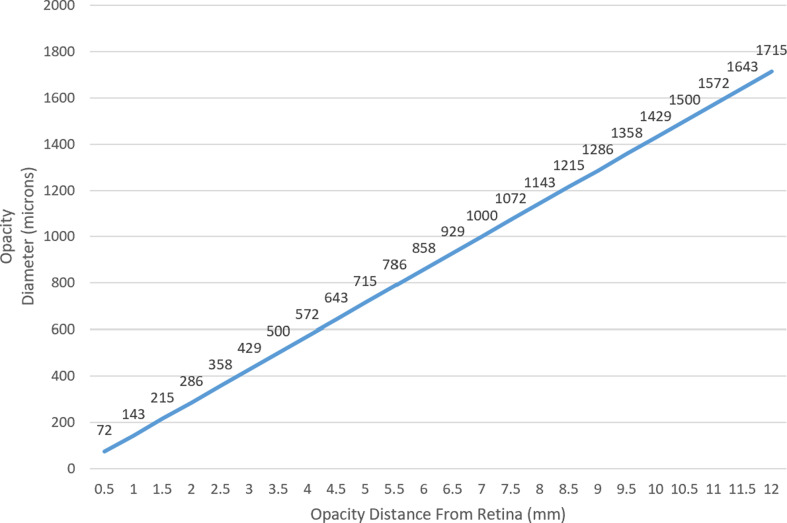
Minimal Opacity Diameter (micron) needed to cast an umbra on retinal surface in relation to the opacity distance from retina (mm), for a 3 mm pupil diameter

**Fig. 5:**
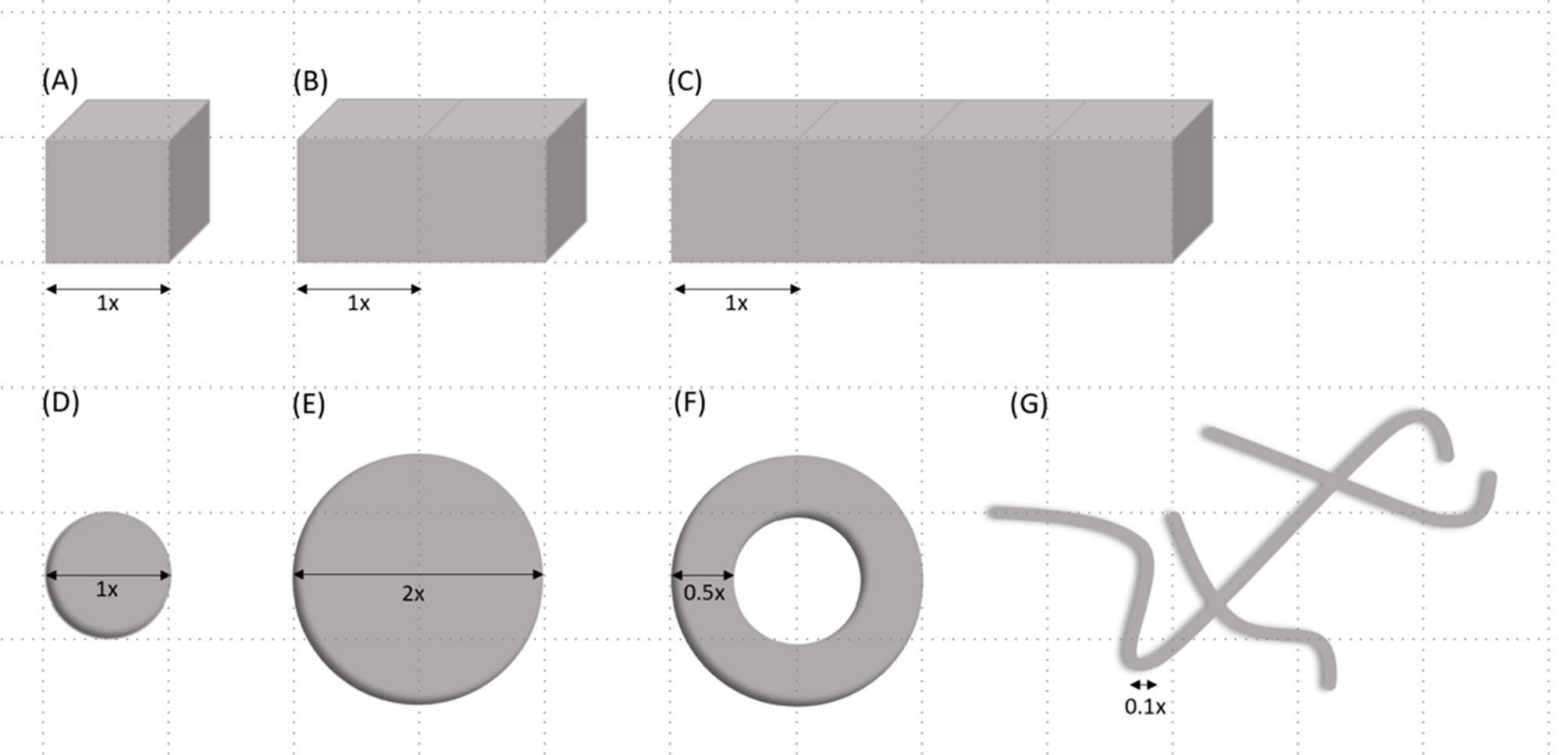
Different opacity shapes and their relevant width/diameter used in calculations. A + B + C: Cube shaped opacities. D + E: Sphere shaped opacities. F: Ring shaped opacity. G: Floater shaped opacity

**Fig. 6:**
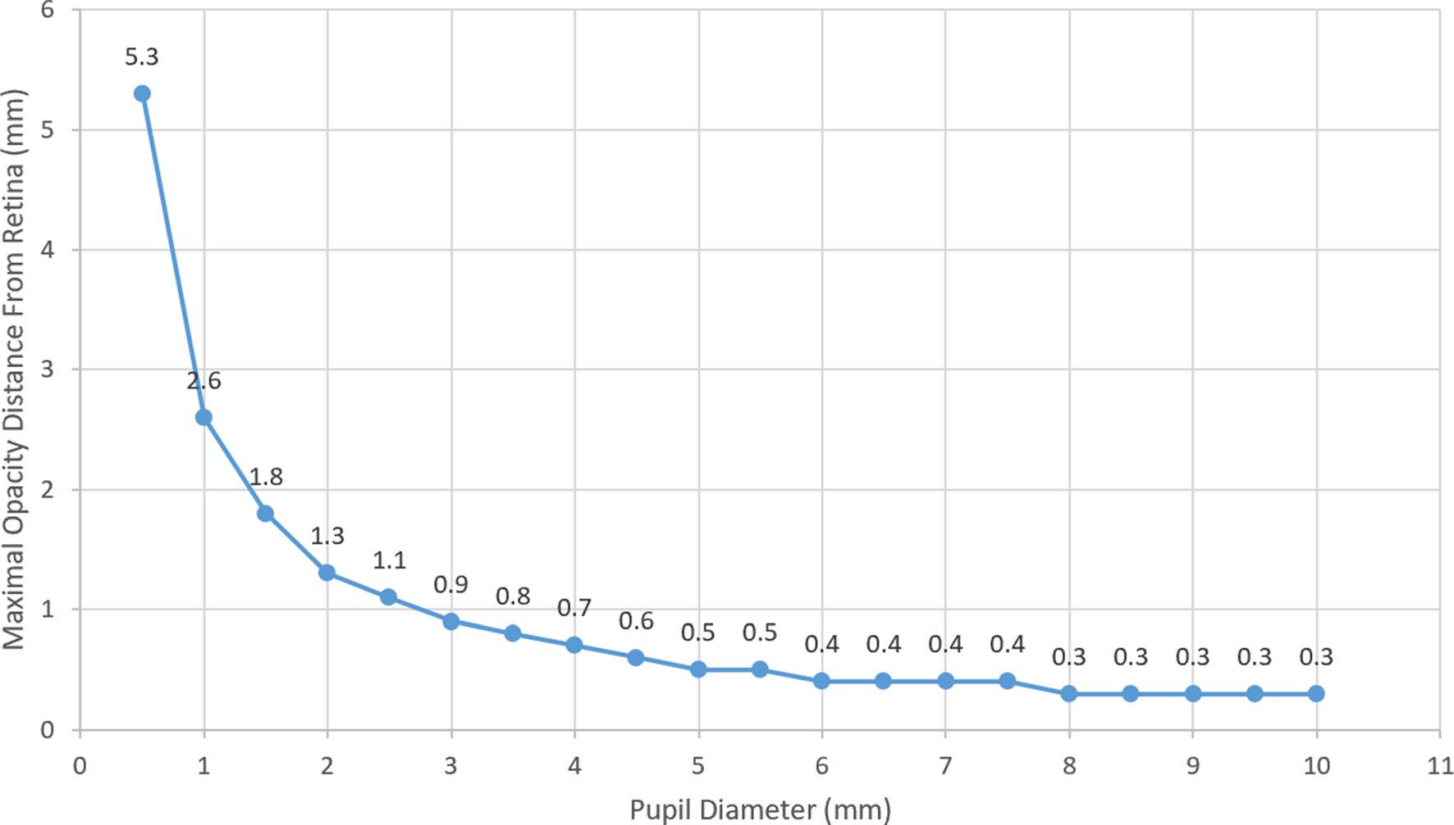
Maximal possible distance from the retina (mm) for an opacity with a diameter of 125 microns to be situated and cast a full retinal shadow in relation to pupil diameter (mm)

Using the above model and Eq. 1, we calculated the minimal opacity diameter needed for a vitreous opacity situated at various distances from the retina to cast a full shadow on the retina (Fig. 4). For an opacity situated in the posterior vitreous, 1.5 mm from the retina, a minimum diameter of 215 microns was needed to cast a full shadow. It is important to note that in the case of a line-like opacity, whether linear or circular (e.g., Weiss ring), the relevant diameter is the width of the thickest part of the opacity, rather than its overall or largest diameter (Fig. 5). In the case of a solid opacity, the shortest axis should be used as the diameter of the opacity. Using Eq. 2 and assuming a 125-micron opacity diameter (largest estimated AH bodies diameter [1, 4]), we calculated the maximal possible distance from the retina for the opacity to be situated while still casting a full shadow on the retina (Fig. 6). In eyes with a pupil diameter of 2 mm, a 125-micron opacity situated further than 1.3 mm from the retina is not expected to cast a full shadow on the retina.

**Fig. 7:**
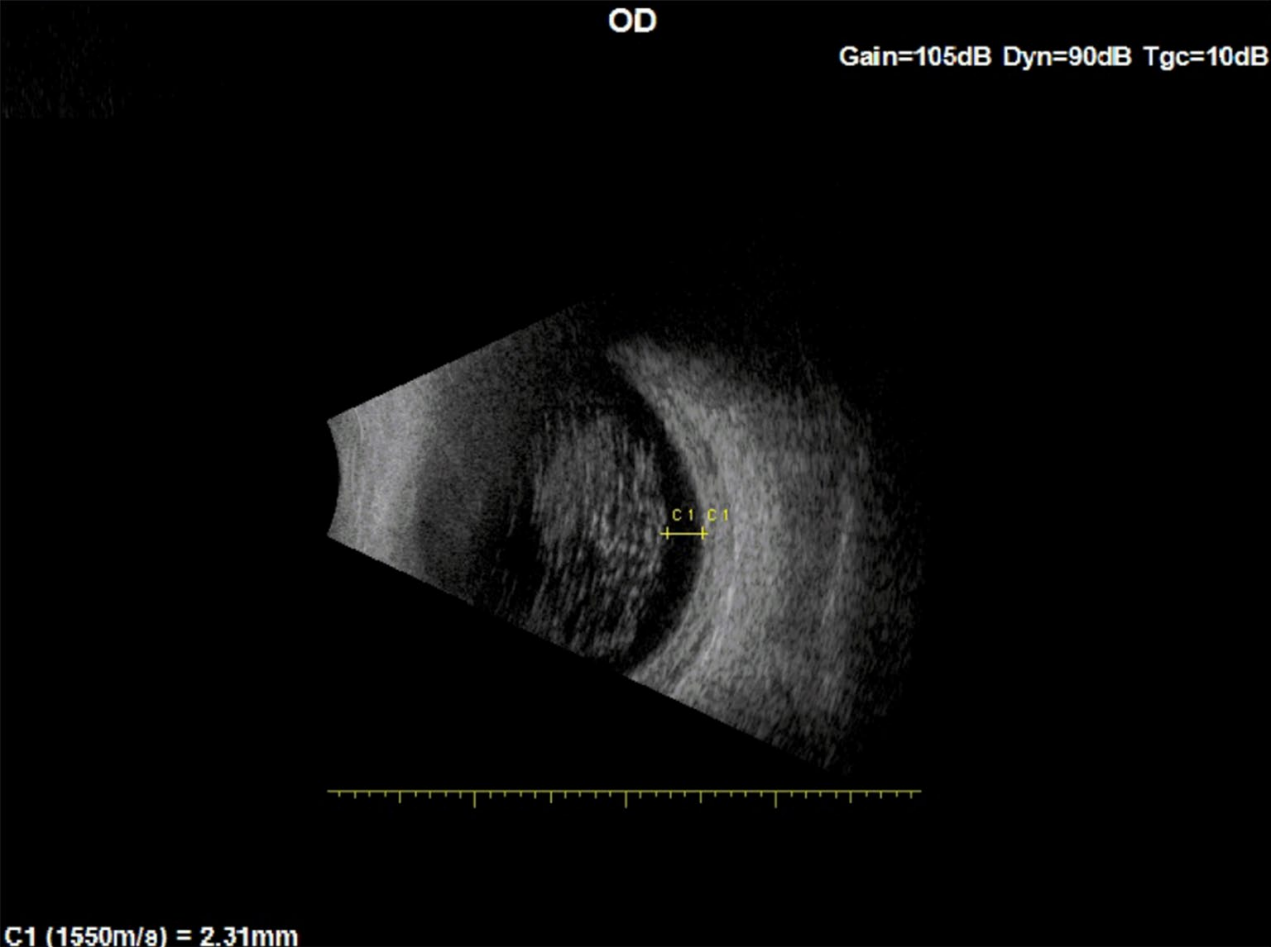
Ultrasound B-mode scan of patient 1 Right Eye. C1: measurement of the closest AH body to distance from the retina

**Fig. 8:**
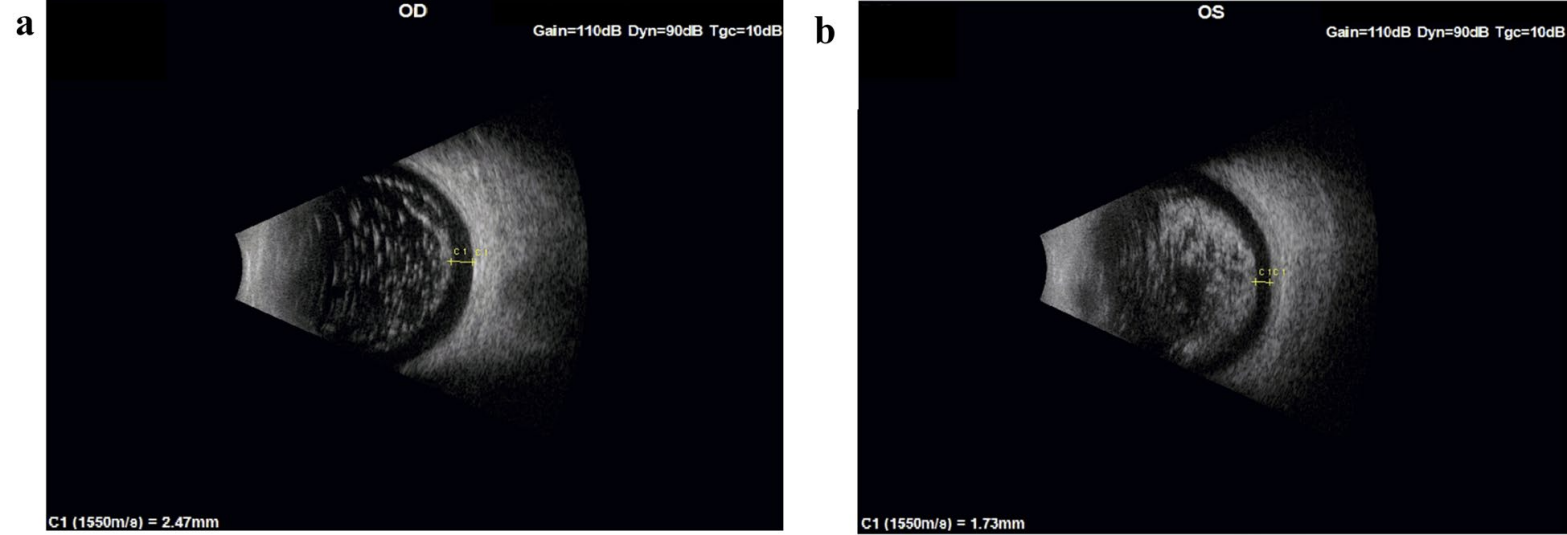
(**a**) Ultrasound B-mode scan of patient 2 Right Eye. C1: measurement of the closest AH body to distance from the retina. (**b**) Ultrasound B-mode scan of patient 2 Left Eye. C1: measurement of the closest AH body to distance from the retina

Measured by ultrasound B-mode scan, the distances of the closest AH body to the retina in the two patients were 2.3 mm, 2.47 mm, and 1.73 mm (Patient 1: Fig. 7, Patient 2: Figure 8a and b).

Combining these measurements with our calculated results (Eq. 1, Fig. 4), in order to cast a retinal shadow, AH bodies situated at the furthest periphery of the vitreous (closest to the retina) in these two patients would need to have a diameter *≥* 330, *≥* 353 microns and *≥* 248 microns, respectively.

## Discussion

AH is a benign degenerative condition characterized by the presence of multiple small yellow-white refractile spheres in the vitreous body [1, 4, 5]. Despite their density, refractivity, and mobility, they rarely cause any visual symptoms [1, 4, 6, 14]. 

In this study, relying on a published model of floater perception, geometry (triangle similarity rules), and B-mode Ultrasound imaging, we proposed the following explanation for this apparent paradox [10, 11, 22, 24]. Since AH bodies are typically not found in the peripheral 1.5 mm of the vitreous [10, 12, 13] (Figs. 7 and 8a and b) and considering their maximal size [1, 4], the shadow they cast does not reach the retina. Furthermore, we demonstrated that under the above conditions, the opacity will not be noticed even with a 2 mm pupil [1, 5, 10, 12, 13]. 

**Fig. 9:**
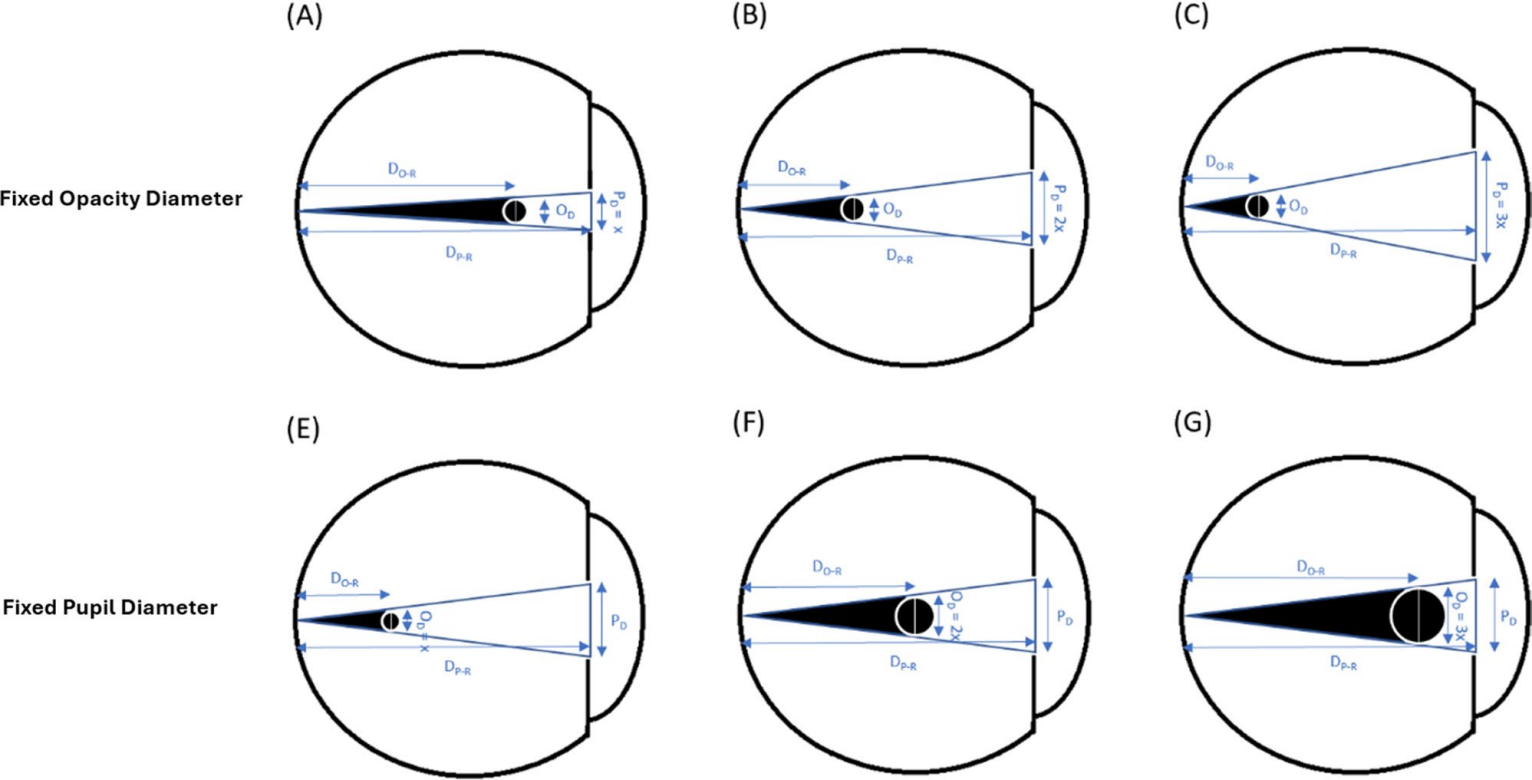
Required opacity position to cast a pinpoint retinal shadow in relation to varying pupil and opacity diameters. Fixed Opacity Size: In diagrams A, B, and C, the opacity diameter remains constant while the pupil diameter increases. Fixed Pupil Diameter: In diagrams E, F, and G, the pupil diameter remains constant while the opacity diameter increases. O_D_: opacity diameter, D_P−R_: distance between pupil and retina, D_O−R_: distance between opacity and retina

Of interest, this model also clearly explains why floaters are more easily perceived in bright light. This can be attributed not only to the greater contrast against which the floater is perceived, but also as in bright light the pupil constricts considerably. Furthermore, for a floater or opacity, such as a dropped lens fragment, to be noticeable when residing at the very center of an emmetropic eye (10.5 mm from the retina), its shortest diameter would have to be 1500 micron, the size of an optic disc. Only larger vitreous opacities would be detected if anteriorly situated (Fig. 9).

This model has direct clinical implications on the surgical management of patients with symptomatic myodesopsia. As shown by our calculations, symptomatic floaters must be either very large and/or situated very close to the retina; hence these are not the floaters procedures such as YAG laser vitreolysis are searching and aiming for [11, 20]. 

Therefore, we propose that in successful cases, the effectiveness of YAG laser vitreolysis is likely due to a mechanism different than the one claimed — that larger floaters are fragmented into multiple smaller ones and displaced out of the visual axis [11, 20]. The alternative mechanism we propose is that the YAG laser energy, aimed towards the center of the vitreous cavity, generates a shock wave that displaces the actual symptomatic floaters that are in close proximity to the retina surface, further away from the retina. Hence, performing YAG laser vitreolysis does not require the surgeon aim at any visible floater, but instead hope for an indirect shock wave mechanism that will displace the symptomatic floaters situated in the outer 1.5 mm of the vitreous body. Further research is necessary to provide stronger evidence supporting the proposed mechanism of YAG laser vitreolysis.

Several limitations of this study need to be considered. First, the study relied on a mathematical model that assumed a fixed pupil to retina distance of 21 mm in an emmetropic eye, while axial lengths vary considerably among individuals [25]. Additionally, each of our analyses required fixing one of the parameters, either the pupil diameter or opacity diameter. The existence of a “clear zone” between the most posterior AH body and retina was inferred from both literature and our clinical observation (Figs. 7 and 8a and b) [10, 12, 13]. AH bodies size was based on literature [1, 4, 5]. As our model shows, larger opacities that are situated closer to the retina could still cause a shadow on the retina. Lastly, based on the literature, we assumed a smooth spherical morphology for AH bodies [1, 15]. If AH bodies have an irregular shape, it could lead to light scattering and optical aberrations, which might affect visual acuity [1, 15]. 

In conclusion, the results of this study provide an interesting and plausible explanation for the rarity of visual symptoms associated with AH. They also suggest a potentially important clinical implication related to the mechanism of YAG laser viterolysis and provide a basis for further research into management of symptomatic vitreous opacities.

## Electronic supplementary material

Below is the link to the electronic supplementary material.

Supplementary Material 1

## Data Availability

No datasets were generated or analysed during the current study.
