# Supplementary material for: The lack of floater perception in eyes with asteroid hyalosis and its direct implications on laser vitreolysis
Source: Int J Retina Vitreous. 2024 Oct 24;10:80. doi: 10.1186/s40942-024-00601-0 (PMC11515566; doi:10.1186/s40942-024-00601-0)
Supplement: Supplementary file 1 — Supplementary Material 1 [file 40942_2024_601_MOESM1_ESM.docx]

June 29, 2023

Dear Dr. Zaher,

Considering your request for exemption of submission to the IRB of your paper titled “The Lack of Floater Perception in Eyes with Asteroid Hyalosis and its Direct Implications on Laser Vitreolysis”, describing 2 unidentified patients together with 3 unidentified eye pictures (enclosed), we hereby provide exemption of submission of this type of research and paper to our local IRB committee at Rambam Health Care Campus.

Sincerely,
Shimon Pollack, MD

Prof Shimon Pollack , MD
Hospital Management
Chairman of Internal Review Board
Institute for Allergy, Immunology & AIDS
Rambam Medical Center
Haifa , Israel
Tel 972-4-7772551
Fax 972-4-7772124
cell 972-50-2062021
[e-mail:pollack@rambam.health.gov.il](mailto:e-mail%3Apollack@rambam.health.gov.il)
